# Is there relationship between epicardial fat and cardiovascular parameters in incident kidney transplant patients? A post-hoc analysis

**DOI:** 10.1371/journal.pone.0191009

**Published:** 2018-02-21

**Authors:** Daniel Constantino Yazbek, Aluizio Barbosa Carvalho, Cinara Sa Barros, Jose Osmar Medina Pestana, Carlos Eduardo Rochitte, Raul Dias dos Santos Filho, Maria Eugênia F. Canziani

**Affiliations:** 1 Nephrology Division, Federal University of Sao Paulo, São Paulo, Brazil; 2 Cardiovascular Magnetic Resonance and Computed Tomography Sector, Heart Institute (InCor), University of São Paulo, Medical School, São Paulo, Brazil; 3 Lipid Clinic Heart Institute (InCor) University of Sao Paulo Medical School, São Paulo, Brazil; Hospital Universitario de la Princesa, SPAIN

## Abstract

**Background:**

Epicardial fat (EF) has been related to increased cardiovascular risk in chronic kidney disease patients. Kidney transplantation is associated with weight gain, especially within the first 12 months. Recently an association between EF and left ventricular mass (LVM) has been suggested in kidney transplant (KTX) recipients.

**Objective:**

Evaluate the EF in KTX recipients and its association with cardiovascular parameters in a 12-month follow-up study.

**Methods:**

EF volume was determined using thoracic computed tomography. The EF progressor group (EF gain) was defined by any increment in EF after 12 months. LVM and LVM index were calculated by echocardiography.

**Results:**

Ninety-eight incident KTX patients [57% men, 41.2 ± 10.1 years, mean dialysis time prior to transplant of 24 (11–60) months] were analyzed. At baseline and after 12 months, EF was 318.6 (275.2–392.6) ml and 329.5 (271.7–384.8) ml, respectively (p = 0.03). When compared to patients who EF decreased (n = 33), those with EF gain (n = 65) had a greater increase of body mass index, abdominal circumference and blood glucose. These patients also had a lower reduction of LVM index. However in the multivariate analysis, there was no difference in LVM index change between groups (interaction p = 0.565), even after adjustment for hypertension, glucose and coronary calcium score (interaction p = 0.538).

**Conclusion:**

The impact of EF gain on ventricular mass after KTX could not be definitely confirmed. Further prospective studies in a large sample of KTX patients should be considered to address a possible causal relationship between EF gain and cardiac hypertrophy in this population.

## Introduction

Obesity is a public health problem that affects a large proportion of the global population and has shown an increasing prevalence in recent decades. It is estimated that > 50% of the US population would be diagnosed with obesity by 2030 [1]. Recent data show that 1 in every 5 Brazilians is diagnosed with obesity, and the obesity rate has increased by approximately 60% in the last 10 years [2]. Obesity is also common in patients diagnosed with chronic kidney disease (CKD), and previous Brazilian reports have shown that 40% of adults with pre-dialysis CKD were overweight and 18% presented with obesity [3]. Among those awaiting a kidney transplant from deceased donor, 23% were diagnosed with obesity, and 2.1% of these patients presented with severe obesity [body mass index (BMI) > 40] [4].

Weight gain is observed in approximately one-third of patients after kidney transplantation (KTX) [5]. This finding is primarily associated with increasing age and the use of corticosteroids [6,7]. Ducloux et al. have reported a mean elevation of 2.7 ± 5.8 kg in patients a year after undergoing KTX [8]. The increase in body weight observed in this population is associated with the occurrence or worsening of metabolic syndrome, hypertension, post-transplant diabetes mellitus, graft loss, cardiovascular diseases, and increased mortality [8–12].

Epicardial fat (EF), a component of the visceral fat compartment, plays a key role in several important physiological functions such as regulation of homeostasis, providing a local energy source, angiogenesis, coronary remodeling, and buffering of the coronary arteries against torsion induced by myocardial contraction [13,14]. EF is known to serve as an endocrine organ with both, local and systemic functions, associated with the secretion of inflammatory hormones and cytokines [14]. An increase of EF has been shown to be associated with the deregulation of pro- and anti-inflammatory cytokines resulting in a state of chronic inflammation, which contributes to the onset and progression of metabolic and cardiovascular disorders [15]. Several studies demonstrate an association between EF and the occurrence of cardiovascular diseases in the general population [16–18]. Recently, cross-sectional studies have demonstrated an association between EF and increased left ventricular mass (LVM) in patients newly diagnosed with systemic arterial hypertension[18] and those diagnosed with CKD including a KTX group [19]. However, no studies have prospectively evaluated the role of EF in the KTX population and its association with cardiovascular disease.

## Objective

We aimed to evaluate the role of EF in KTX patients and its association with cardiovascular parameters by performing a 12-month follow-up study.

## Materials and methods

This is a post-hoc analysis of a randomized, controlled, and open-label study that evaluated the effects of statin use on coronary artery calcification in 100 incident KTX recipients [20].

### Study population

Recipients of KTX who were within 60 days post-transplant procedure were considered suitable to participate in the study. Exclusion criteria were: age < 18 or > 60 years, creatinine clearance < 30 mL/min, patients prioritized to undergo KTX, and those who had presented with any cardiovascular event or received statin 3 months prior to transplantation. Imaging studies could not be evaluated in 2 patients; thus, EF measurements were analyzed in 98 patients.

According to the local protocol, all of the patients underwent initial immunosuppression with prednisone. Seventy-nine patients (66%) were KTRs of living donors, whereas those with totally matched human leukocyte antigen (HLA) were 22%, partially matched 35% and fully mismatched 8%. KTRs of living donors with totally matched received cyclosporine and azathioprine and partially or fully mismatched used tacrolimus and azathioprine. Of note, preemptive transplantation was performed only in one patient. The KTRs of deceased donors were induced with basiliximab and received tacrolimus and mycophenolic acid. Thymoglobulin was used when patients had a higher panel reactive antibody (> 20%). No patients were using vitamin K antagonist during the study.

The study was reviewed and approved in 02/03/2007 by the Ethics Advisory Committee of the Federal University of São Paulo, and each patient signed the written informed consent form. Important to note, no deviation occurred in the duration of the study protocol. This study was registered at The Brazilian Clinical Trials Registry (REBEC—www.ensaiosclinicos.gov.br/) after the beginning the study, under the RBR-32RFMB number. The delay in registration was because at the time of the beginning of the study the REBEC platform was not available.

In addition, the authors confirm that all ongoing and related trials for this drug/intervention are registered.

### Laboratory data

Fasting blood samples were drawn to determine the following: serum creatinine, cystatin C, serum glucose, lipid profile, pH, bicarbonate, and C-reactive protein (CRP, immunometric assay, Immulite). Creatinine clearance was estimated using the Chronic Kidney Disease-Epidemiology Collaboration (CKD-EPI) creatinine/cystatin C equation [21].

### Assessment of images

Patients underwent thoracic computed tomography to obtain EF and coronary calcium score measurements. Previously obtained images were re-evaluated to measure EF using a Vitrea Core Enterprise Suite workstation-VES (Vital Images Inc., Plymouth, MN, USA). These were electrocardiogram-triggered axial images of the thorax covering the entire heart and acquired using standard parameters described elsewhere [22]. The regions of interest located around the heart on its epicardial surface showing a density between -30 and -200 Hounsfield units were defined as fat. EF located on the inner pericardial surface, which was in direct contact with the epicardial surface of the heart, was demarcated by drawing a line along the pericardial path. After defining the regions of interest, a volumetric tool was used to measure the mean density of the pixels in each region of interest, as well as the volume of each compartment (expressed as mL). The evolution of EF over time was calculated based on the volumetric variation in pericardial fat at baseline and after 12 months (delta = 12 months–baseline epicardial fat). The progressor fat group showed a delta EF > 0.

The coronary calcium score was determined by multiplying the area of each calcified lesion by a weighting factor corresponding to the peak pixel intensity for each lesion. The sum of each lesion of all coronary arteries was considered for the analysis as previously described [22]. The calcium score was expressed as Agatston Units (AU), and the presence of coronary calcification was defined as a calcium score > 10 AU.

Two-dimensional color Doppler echocardiography (Philips HDI 5000, Royal Philips Electronics, Netherlands) was performed based on the recommendations of the American Society of Echocardiography [23]. The left atrial diameter (LAD), diameter of the interventricular septum (SIV), left ventricular posterior wall thickness (LVEP) and left ventricular diastolic diameter (LVD) were measured in millimeters. Using the Teichholz method, we analyzed left ventricular systolic function based on the ejection fraction. The left ventricular mass (LVM) and left ventricular mass index (LVMI) were calculated based on the formulas: LVM (g) = 0.8 X (1.04 X [LVD + SIV + LVEP]^3^ - [LVD]^3^) + 0.6 and LVMI = LV mass/body surface area. A value of LVMI > 115 g/m^2^ for men and > 95 g/m^2^ for women was interpreted as the presence of left ventricular hypertrophy.

### Statistical analysis

The mean and standard deviation, median and interquartile range, or frequency (proportion) were calculated for the variables. The Kolmogorov-Smirnov statistical test was used to investigate the normal distribution of data. Comparisons of continuous variables between groups were performed using the Student’s t-test and the Mann-Whitney U-test, and within groups using the Student’s t-test or Wilcoxon signed rank test for normal and skewed data, respectively. Comparisons of proportions were performed using chi-squared analysis or the Fischer exact test or the McNemar test. The generalized estimating equation (GEE) was used to identify the association between the changes of EF and LMVI. The final model was adjusted for hypertension, glucose and coronary calcium score. P values < 0.05 were considered statistically significant. All statistical analyses were performed using SPSS for Windows version 15.0 software (SPSS Inc, Chicago, IL).

## Results

Baseline characteristics of the study population are shown in Table 1. Patients were between 20 and 60 years of age, predominantly men, with a high prevalence of hypertension and a sedentary lifestyle. The mean prior dialysis time was 2 years and 66% of the patients had received live donor kidneys. We observed that 30% of the patients were overweight, 8% had been diagnosed with obesity, and 40% showed an increased abdominal circumference. We observed that 62% of the patients were stage 3 CKD, 19% showed fasting serum glucose > 100 mg/dL, 55% showed elevated low-density lipoprotein (LDL)-cholesterol and 52% showed hypertriglyceridemia. Regarding cardiovascular parameters, 20% of the patients showed an increased LAD, 5% showed decreased left ventricular ejection fraction, 64% showed left ventricular hypertrophy, and 33% showed coronary calcification.

**Table 1:** Characteristics of the study population at baseline (n = 98).

**age (years)**	41.2 ± 10.1
**Male n (%)**	56 (57)
**Smoking n (%)**	8 (8)
**Hypertension n (%)**	87 (89)
**Diabetes n (%)**	7 (7)
**Sedentary n (%)**	77 (78)
**Prior dialysis time (months)**	24(11–60)
**Deceased donors n(%)**	33 (34)
**Use of statins n (%)**	50 (51)
**BMI (kg/m^2^)**	23.9 ± 4.1
**WC (cm)**	87.7 ± 10.9
**SBP (mmHg)**	131.5 ± 14.7
**DBP (mmHg)**	83.4 ± 10.0
**CKD EPI Cr/Cys (ml/min/1.73 m2)**	47.5 ± 12.9
**Glucose (mg/dl)**	88 (80–97)
**Total cholesterol (mg/dl)**	201.9 ± 42.4
**HDL-c (mg/dl)**	55.4 ± 15.8
**LDL-c (mg/dl)**	110.8 ± 32.4
**Triglycerides (mg/dl)**	153 (109–153)
**C-reactive protein (mg/l)**	0.09 (0.04–0.33)
**Left atrium (mm)**	38.2 ± 5.1
**Fraction ejection**	0.68 (0.64–0.71)
**LVM index (g/m**^**2**^**)**	123 (98–159)
**Coronary calcium score (AU)**	0 (0–24) 132.9 ± 50.9

Mean ± standard deviation, median (interquartiles)

BMI—body mass index; WC—waist circumference; SBP—systolic blood pressure; DBP—diastolic blood pressure; HDL-c—HDL cholesterol; LDL-c—LDL cholesterol; LVM—left ventricular mass

**Fig 1:**
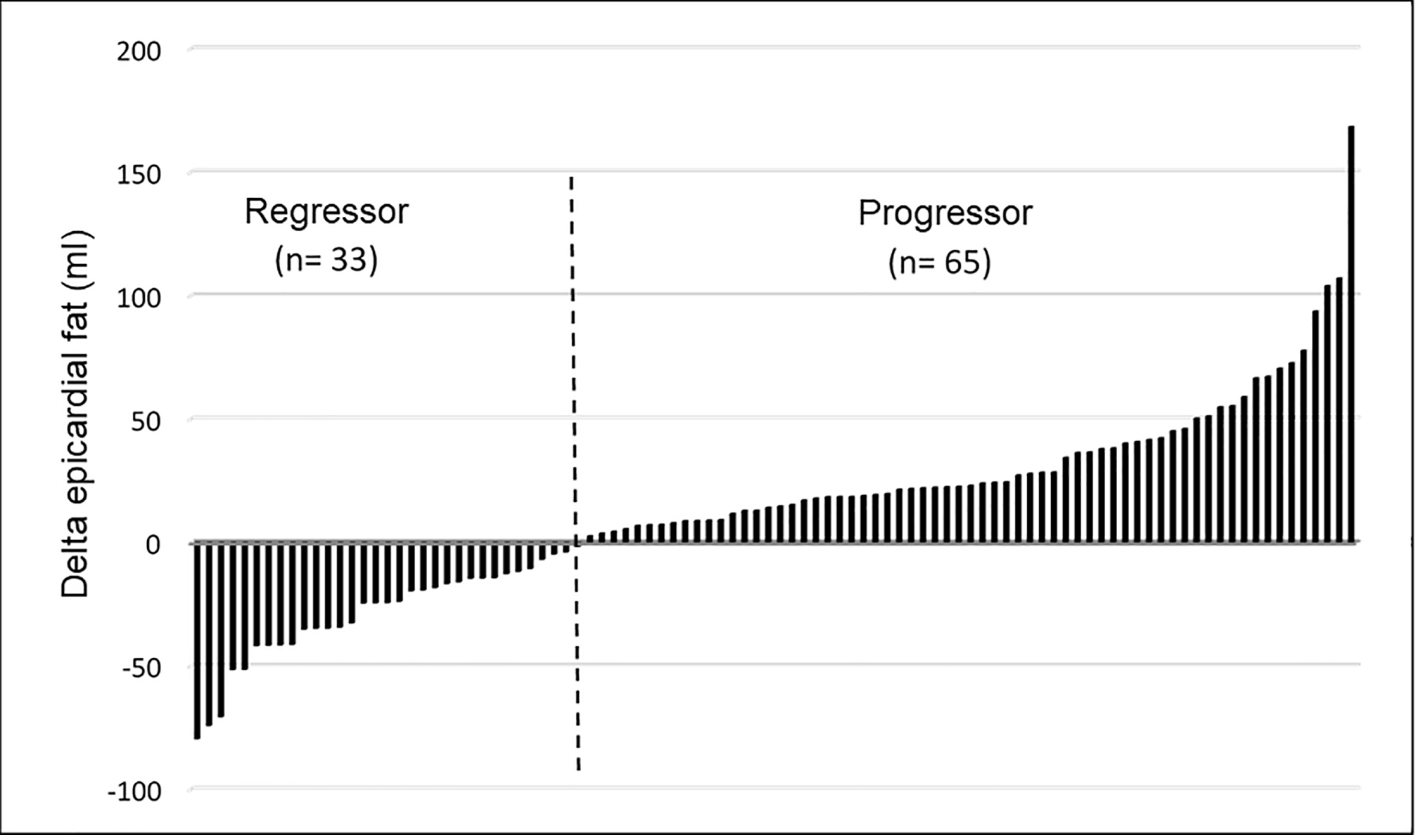
Behavior of epicardial fat (EF) during the study.

The EF volume was 318.6 mL (275.2–392.6) and 329.5 mL (271.7–384.8), at baseline and after 12 months, respectively (P = 0.03). The median change in EF was 9.12 mL (-14.5 to 28.2). In 33 patients the EF volume was observed to decrease, while in 65 patients the EF value was observed to increase during the course of 12 months (fat regressor and fat progressor groups, respectively, Fig 1).

Table 2 shows demographic and laboratory characteristics of patients at baseline and at 12 months based on EF groups. During the study, patients from the fat regressor group showed an increase in BMI, bicarbonate, CRP, and a decrease in total cholesterol, high-density lipoprotein (HDL), LDL, and triglycerides. Patients from the fat progressor group showed an increase in BMI, waist circumference, serum glucose, pH, bicarbonate and a decrease in total, HDL and LDL-cholesterol.

**Table 2:** Comparison of demographic and laboratory parameters in the epicardial fat progressor and regressor groups.

	Progressor (n = 65)			P	Regressor (n = 33)			P	P between groups		
	Baseline	12 month	Delta		Baseline	12 month	Delta		Baseline	12 month	Delta
**Age (years)**	41.3 ± 10.6				41.4 ± 9.2				0.90		
**Male n (%)**	35 (54)				21 (64)				0.35		
**Smoking n (%)**	4 (6)				4 (12)				0.44		
**Hypertension n (%)**	59 (91)				28 (85)				0.50		
**Diabetes n (%)**	6 (9)				1 (3)				0.42		
**Sedentary n (%)**	51 (78)				26 (79)				0.97		
**Prior dialysis time (months)**	24 (11–59)				24 (12–61)				0.66		
**KTXRs with deceased donors n (%)**	46 (71)				19 (58)				0.19		
**Use of statins n (%)**	34 (52)				16 (48)				0.72		
**BMI (kg/m**^**2**^**)**	24.0 ± 4.3	26.7 ± 4.9	2.67 ± 2.62	< 0.001	23.7 ± 3.7	25.1 ± 4.6	1.33 ± 1.92	< 0,001	0.71	0.11	0.005
**WC (cm)**	87.2 ± 11.7	94.1 ± 12.7	7.69 ± 9.63	<0.001	88.8 ± 9.2	90.6 ± 10.3	1.74 ± 5.55	0.08	0.45	0.15	<0.001
**SBP (mmHg)**	131.7 ± 15.3	130.0 ± 14.2	-1.7 ±18.6	0.47	131.2 ± 13.9	130.6 ± 13.2	-0.6 ±18.2	0.85	0.88	0.83	0.78
**DBP (mmHg)**	82.9 ± 10.1	82.8 ± 8.9	-0.15 ±12.4	0.92	84.2 ± 10.0	83.0 ± 8.1	-1.2 ±12.7	0.59	0.54	0.88	0.69
**CKD EPI Cr/Cys (ml/min/1.73 m**^**2**^**)**	47.7 ± 12.6	50.5 ± 12.5	2.8 ±10.8	0.08	47.1 ± 13.7	51.6 ± 15.4	3.6 ±11.1	0.13	0.85	0.75	0.77
**Glucose (mg/dl)**	87 (79–94)	92 (82–99)	6.0 (-3.0–12.5)	0.03	91 (80–99)	89 (84–97)	1.0 (-8.5–6.5)	0.45	0.20	0.77	0.03
**Total cholesterol (mg/dl)**	201.8 ± 41.4	170.2 ± 38.3	-32.6 ±46.1	<0.001	202.1 ± 44.9	160.4 ± 36.6	-41.6 ±42.1	<0.001	0.98	0.22	0.30
**HDL-c (mg/dl)**	56.3 ± 14.9	46.1 ± 13.3	-10.2 ±14.4	<0.001	53.7 ± 17.7	46.0 ± 15.6	-7.7 ±12.8	0.002	0.46	0.96	0.40
**LDL-c (mg/dl)**	112.9 ± 33.0	98.0 ± 32.8	-16.7 ±32.9	<0.001	106.4 ± 31.1	83.6 ± 31.2	-22.3 ±26.2	<0.001	0.35	0.04	0.41
**Triglycerides (mg/dl)**	141 (105–196)	134 (81–189)	-12 (-62–29)	0.20	171 (117–231)	139 (111–186)	-32 (-99–35)	0.05	0.10	0.28	0.24
**pH**	7.30 ± 0.06	7.32 ± 0.05	0.03 ±0.05	<0.001	7.30 ± 0.05	7.33 ± 0.04	0.02 ±0.06	0.10	0.79	0.51	0.55
**Bicarbonate (mmol/l)**	26 (22–28)	27 (25–29)	1 (-1–4)	0.003	24 (20–26)	26 (24–28)	2 (-1–5)	0.02	0.04	0.08	0.74
**C-reactive protein (mg/l)**	0.10 (0.04–0.34)	0.18 (0.09–0.35)	0.03 (-0.19–0.14)	0.71	0.09 (0.04–0.23)	0.27 (0.08–0.76)	0.10 (-0.03–0.40)	0.008	0.83	0.30	0.07

Mean ± standard deviation, median (interquartiles)

KTXR—kidney transplant recipients; BMI—body mass index; WC—waist circumference; SBP—systolic blood pressure; DBP—diastolic blood pressure; HDL-c—HDL cholesterol; LDL-c—LDL cholesterol.

Comparing between the groups we observed that the fat progressor group showed higher bicarbonate levels at baseline and higher LDL-cholesterol levels at 12 months. This group also showed a greater increase in BMI, waist circumference and glucose levels during the study. No statistically significant difference was observed between the fat progressor and regressor groups in terms of the use of antihypertensive drugs, such as beta (β) blockers (46 vs. 48%, p = 0.83), calcium channel blockers (40 vs. 39%, p = 0.95), angiotensin converting enzyme inhibitors/angiotensin II receptor blockers (ACEI/ARB) (29 vs. 30%, p = 0.91). Of note, there were no difference in occurrence of acute rejection between the groups (8% in progressor vs. 9% regressor groups, p = 1.0).

**Fig 2:**
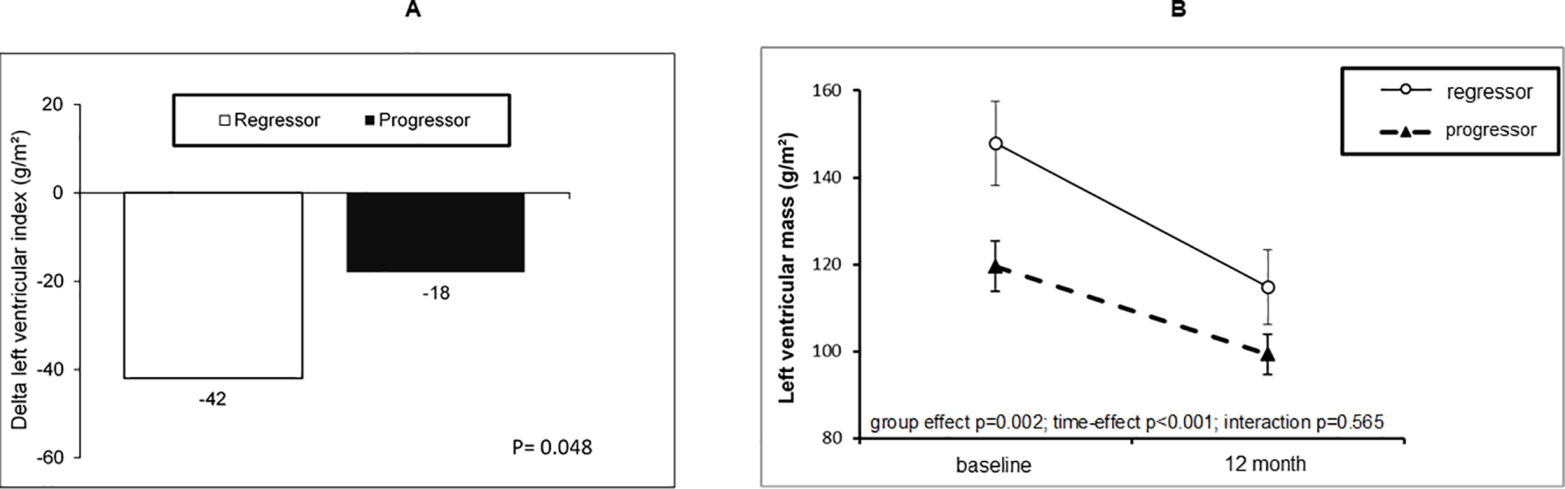
Comparison of the left ventricular mass index during the study between the epicardial fat progressor and regressor groups (**2A** –delta of LVMI and **2B** –GEE model).

Table 3 shows the comparison of cardiovascular parameters between the groups. LVMI was observed to decrease in both groups during the study. A significantly smaller decrease in the LVMI was observed in the fat progressor group than in the fat regressor group (p = 0.048; Fig 2A). Additionally, a decrease of ≥ 30% in the LVMI was observed in 16% and 44% of the patients in the fat progression and regression groups, respectively (p = 0.043). There were no statistically significant differences observed between the groups in terms of changes in the left atrium diameter, ejection fraction, and coronary calcium score.

**Table 3:** Comparison of cardiovascular parameters in the epicardial fat regressor and progressor groups.

	Progressor (n = 65)			P	Regressor (n = 33)			P	P between groups		
	Baseline	12 month	Delta		Baseline	12 month	Delta		Baseline	12 month	Delta
**Epicadial Fat (ml)**	309.7 (257.3–354.5)	341.0 (282.5–386.8)	23.2 (14.5–43.9)	<0.001	337.2 (295.6–415.6)	314.2 (266.3–390.2)	-24.4 (-41.4 –-14.4)	<0.001	0.02	0.42	<0.001
**Left atrium (mm)**	37.8 ± 5.4	36.6 ± 4.5	-0.8 ±4.4	0.30	38.9 ± 4.8	38.2 ± 5.2	-0.8 ±5.2	0.57	0.36	0.24	0.99
**Ejection fraction**	0.69 (0.66–0.72)	0.67 (0.64–0.73)	-0.01 (-0.04–0.04)	0.53	0.65 (0.60–0.70)	0.68 (0.65–0.75)	0.04 (-0.01–0.05)	0.13	0.02	0.71	0.11
**LVM index (g/m^2^)**	109 (87–137)	92 (75–115)	-18 (-35 –-2)	0.001	136 (106–175)	107 (76–141)	-42 (-51 –-17)	0.002	0.009	0.14	0.048
**Coronary calcium score (AU)**	0 (0–75)	0 (0–107)	0 (0–1)	0.56	0 (0–77)	3 (0–172)	0 (0–23)	0.005	0.53	0.18	0.07

Mean ± standard deviation, median (interquartiles)

LVM—left ventricular mass.

The multivariate model GEE (Fig 2B) shows that there was no statistically significant difference in the LVMI between groups (group effect p = 0.002, time effect p < 0.001, interaction p = 0.565) even after adjustment for hypertension, serum glucose, and coronary calcium score (group effect p = 0.024, time effect p < 0.001, interaction p = 0.538).

## Discussion

In this prospective study an increase in EF volume was observed in 65%, while a decrease of LVM was observed in 79% of the incident KTX recipients, after a 12-month follow-up. No relationship between the change of EF and LVM could be definitively confirmed.

Obesity, represented by elevation of waist circumference and BMI, is a common finding in early post-transplant recipients and has been associated with an increased risk of cardiovascular disease and graft failure [24]. A recent study demonstrated a progressive elevation in BMI during a 12-month follow up after KTX [25]. Hoogeveen et al. have reported an increase in the prevalence of obesity (5.6% to 11.4%) a year after KXT [9]. In the present study a significant increase in BMI and 50% increase in the prevalence of obesity was observed after 12 months (8–20%, data not shown). This weight gain could be related to an increased appetite secondary to steroid therapy, improvement of the uremic milieu, and an initial period of physical inactivity following transplant surgery. Post-transplant weight gain has also been associated with the presence of hyperglycemia and diabetes (28). Data obtained from the Organ Procurement and Transplantation Network and Scientific Registry of Transplant Recipients (OPTN/SRTR) have shown that the incidence of diabetes is higher in those diagnosed with obesity/overweight patients—in fact, within a year, the prevalence of post-transplant diabetes in those with obesity/overweight patients was 10% compared to only 3% in those with a normal BMI [26]. Moreover, the risk of new-onset diabetes mellitus increases linearly with a rise of every 1 kg above 45 kg [27]. Recent studies have suggested that impairment in glucose metabolism secondary to insulin resistance and inflammation could lead to an increase in visceral fat in this population [28,29]. However, in the present study, we observed only a change toward to higher glucose levels but not to inflammation in the progressor group. Unfortunately, the insulin resistance index was not evaluated in this study.

EF is increased and associated with BMI in patients with CKD [30,31]. Recently, Okyay et al. have shown an important relationship between EF and BMI, waist circumference and percentage of body fat mass analyzed by bioimpedance in patients who underwent hemodialysis [32]. In the present study, we prospectively demonstrated a relationship between an increase of EF in parallel with an increase of BMI in incident KTX recipients. Of note, Cordeiro et al. [33] have described a relationship between increases of EF and incidence of cardiovascular events in CKD patients. Additionally, in that study, EF was a better predictor of cardiovascular risk than abdominal visceral fat.

Few studies have evaluated the relationship between EF and LVM in patients undergoing KTX [19]. It is well known that ventricular hypertrophy, which is a common finding in KTX recipients [34] is associated with cardiovascular outcomes [35]. Çolak et al., in a cross-sectional study, have demonstrated a lower EF volume in KTX patients compared to those in hemodialysis. Interestingly, the authors observed a direct relationship between EF volume and LVM in both groups [19]. The pathways which links EF with ventricular remodeling remains unclear. However, a direct influence of fat gain by obstructive factor or paracrine mechanism, via inflammatory mediators, adipokines and activation of the renin angiotensin aldosterone system, could be a possible explanation [36,37]. Although, our results suggest an association between EF gain and lower reduction of VM, the adjusted statistical analysis did not confirm this finding.

We have previously demonstrated an independent relationship between pericardial fat and coronary calcification in pre-dialysis CKD patients [38]. Kerr et al. have demonstrated an association between EF, interleukin 6, and vascular calcification in a similar population [31]. However, no data describe the role of pericardial or EF in vascular calcification in KTX recipients. In the present study, no relationship was observed between the presence or progression of coronary calcification and EF. This result could be attributed to the young age of the study population, short duration of previous dialysis therapy, a low prevalence of diabetic patients, low incidence of coronary calcification, and a well-functioning kidney graft.

To our knowledge, this is the first study to evaluate the behavior of EF in incident KTX. Although some results pointed out a possible relationship between EF and LVM, this finding could not be confirmed. A possible explanation relies on the limitations of this study such as, a relatively small and young group of patients, and a short period of follow-up.

## Conclusion

The impact of EF gain on ventricular mass after KTX could not be definitely confirmed. Further prospective studies in a large sample of KTX patients should be considered to address a possible causal relationship between EF gain and cardiac hypertrophy in this population.

## Supporting information

S1 File Avaliable study data PLOS ONE.xlsx. (XLSX)
